# Analysis of the intestinal microbiota and profiles of blood amino acids and acylcarnitines in neonates with hyperbilirubinemia

**DOI:** 10.1186/s12866-024-03328-y

**Published:** 2024-05-18

**Authors:** Junguo Li, Shenglin Ye, Xinyuan Huang, Guolong Yang, Yijin Wang, Jianghui Zeng, Chunhui Lai

**Affiliations:** 1 Department of Clinical Laboratory, The Second Nanning People’s Hospital, The Third Affiliated Hospital of Guangxi Medical University, Nanning, Guangxi China; 2 Guangxi Key Laboratory of Molecular Immunology Research, Nanning, Guangxi China; 3 Department of Clinical Laboratory, The Fifth Affiliated Hospital of Guangxi Medical University, The First People’s Hospital of Nanning, Nanning, Guangxi China; 4 Department of Pediatrics, The Second Nanning People’s Hospital, The Third Affiliated Hospital of Guangxi Medical University, Nanning, Guangxi China

**Keywords:** Hyperbilirubinemia, Neonates, Intestinal microbiota, Amino acids, Acylcarnitines

## Abstract

**Objective:**

This study aimed to discuss the distinctive features of the intestinal microbiota in neonates with hyperbilirubinemia and to comprehensively analyse the composition of the intestinal microbiota as well as the levels of free amino acids and acylcarnitines in the peripheral blood of neonates experiencing hyperbilirubinemia.

**Results:**

At the phylum level, *Proteobacteria*, *Firmicutes*, *Actinobacteria*, *Bacteroidetes*, and *Chloroflexi* were the five predominant microbial groups identified in both the hyperbilirubinemia and control groups. Alpha diversity analysis, encompassing seven indices, showed no statistically significant differences between the two groups. However, Beta diversity analysis revealed a significant difference in intestinal microbiota structure between the groups. Linear discriminant analysis effect size (LEfSe) indicated a significant reduction in the abundance of *Gammaproteobacteria* and *Enterobacteriaceae* within the hyperbilirubinemia group compared to that in the control group. The heatmap revealed that the control group exhibited increased abundances of *Escherichia* and *Bifidobacterium*, while the hyperbilirubinemia group exhibited increased levels of *Enterococcus* and *Streptococcus*. Regarding blood amino acids and acylcarnitines, there were greater concentrations of citrulline (Cit), arginine (Arg), ornithine (Orn), and valine (Val) in the hyperbilirubinemia group than in the control group. The hyperbilirubinemia group also exhibited significant increases in medium-chain fatty acids (C6, C8), long-chain fatty acids (C18), and free carnitine (C0).

**Conclusion:**

By comparing neonates with hyperbilirubinemia to those without, a significant disparity in the community structure of the intestinal microbiota was observed. The intestinal microbiota plays a crucial role in the bilirubin metabolism process. The intestinal microbiota of neonates with hyperbilirubinemia exhibited a certain degree of dysbiosis. The abundances of *Bacteroides* and *Bifidobacterium* were negatively correlated with the bilirubin concentration. Therefore, the fact that neonates with hyperbilirubinemia exhibit some variations in blood amino acid and acylcarnitine levels may provide, to a certain degree, a theoretical basis for clinical treatment and diagnosis.

**Supplementary Information:**

The online version contains supplementary material available at 10.1186/s12866-024-03328-y.

## Introduction

Neonatal jaundice (NJ), characterized by yellowing of the sclera and skin, is a common clinical condition in the neonatal period. If the level of bilirubin increases very quickly or indicates progressive deterioration, the physiological jaundice may develop to pathological jaundice [1]. Neonatal hyperbilirubinemia (NHB) is diagnosed when the bilirubin level in a newborn’s body continues to increase and reach the criteria for phototherapy [2]. Studies indicate that up to 60% of full-term infants and 80% of preterm infants may experience hyperbilirubinemia in the early neonatal period. Untimely diagnosis and treatment can lead to elevated serum bilirubin levels, causing multiorgan dysfunction in affected infants. Bilirubin can also interfere with the normal metabolism of nerve cells by crossing the blood‒brain barrier, resulting in disturbances in the functioning of the nervous system and, in severe cases, leading to disability or death [3, 4]. Therefore, early identification, diagnosis, and timely intervention are of considerable clinical significance in preventing potential severe complications.

The human intestinal tract harbours trillions of microorganisms, forming a complex ecosystem with extensive metabolic activity collectively referred to as the intestinal microbiota, representing the so-called’second genome’ of humans [5].The human intestinal microbiota is a complex, dynamic, and extensive ecosystem. It is crucial for maintaining internal balance of the host and influences the hosts’ behaviors [6, 7]. The intestinal microbiota plays an essential role in the transformation and metabolism of bilirubin. Upon entering the intestines, certain bacteria in the intestines convert bilirubin to stercobilinogen and promote its excretion by accelerating intestinal motility [8, 9]. Studies have shown that genetically modified mice raised under germ-free conditions exhibit significantly greater serum bilirubin levels than those raised under conventional conditions [10]. Neonates with jaundice may lack certain key bacteria in their intestines, leading to an imbalance in the intestinal microbiota. Changes in the intestinal microbiota have also been observed in jaundiced infants before and after treatment [11]. Consequently, identifying the association between the intestinal microbial community and neonatal hyperbilirubinemia is essential, and identifying differential microbial groups is crucial.

This study employed 16 S rRNA gene sequencing to explore the characteristics of the intestinal microbiota in neonates with hyperbilirubinemia. Furthermore, our objective was to scrutinize and compare the variations in blood amino acid and acylcarnitine levels between neonates with hyperbilirubinemia and their counterparts in the control group. We also aimed to identify potential bacterial markers in the intestinal microbiota associated with hyperbilirubinemia, providing a theoretical foundation for the clinical diagnosis and treatment of this condition.

## Materials and methods

### Study population

The study recruited 14 neonates with hyperbilirubinemia, born between February and September 2022, at the Second Nanning People’s Hospital as the hyperbilirubinemia group. Simultaneously, 14 neonates without hyperbilirubinemia were sampled as the control group. Details of both groups regarding sex, gestational age at birth, birth weight, and total bilirubin (TBIL), direct bilirubin (DBIL), and indirect bilirubin (IBIL) levels are provided in Table 1. The inclusion criteria for neonates were as follows: (1) full-term neonates born through normal delivery; (2) mixed feeding for both groups; and (3) birthweight exceeding 2.5 kg. The exclusion criteria for individuals were as follows: (1) showing meconium-stained amniotic fluid, birth asphyxia, or an average Apgar score or single Apgar score which was less than 7; (2) having a history of long-term probiotic or antibiotic use during pregnancy and lactation; and (3) being neonates with congenital genetic disorders. The diagnostic criteria for hyperbilirubinemia were based on the *Diagnosis and Management of Hyperbilirubinemia in the Newborn Infant 35 or More Weeks of Gestation*, published by the American Academy of Pediatrics [12], wherein bilirubin levels exceeded the 95th percentile of the hour-specific Bhutani nomogram for neonatal bilirubin levels. Control Group: Bilirubin levels did not exceed the 95th percentile of the hour-specific Bhutani nomogram. No blue light therapy and probiotics were given before the sample collection and no neonates had hemolytic disease. This study was reviewed and approved by the Medical Ethics Committee of the Second Nanning People’s Hospital (Ethics Record Number: Y2021001) and was conducted in accordance with the principles of the *Helsinki Declaration*.

**Table 1:** Clinical characteristics of the neonates

Characteristics	Hyperbilirubinemia group	Control group	Statistical value	*p* Value
**n**	14	14		
**Sex**			*X*^2^ = 0.150	0.558
Male	9	8		
Female	5	6		
**Nation**			*X*^2^ = 1.168	0.699
Han zu	5	6		
Zhuang zu	6	7		
Other ethnic minorities	3	1		
**Age, days, mean ± SD**	10.14 ± 1.51	10.00 ± 1.41	t = 0.258	0.798
**Gestational age, weeks, mean ± SD**	39.14 ± 0.87	38.81 ± 0.77	t = 1.062	0.298
**Birth weight, g, mean ± SD**	3148.57 ± 357.06	3142.86 ± 247.87	t = 0.049	0.961
**Apgar score**	9.71 ± 0.55	9.83 ± 0.44	Z =−1.11	0.265
**Serum bilirubin (umol/L)**				
Total bilirubin (TBIL)	218.09 ± 64.32	48.46 ± 28.71	t’ = 9.011	< 0.001
Direct bilirubin (DBIL)	10.21 ± 4.30	6.23 ± 2.32	Z =−2.482	0.013
Indirect bilirubin (IBIL)	207.87 ± 63.29	42.23 ± 29.41	t’ = 8.88	< 0.001

### Total DNA extraction from the microbial community

Neonatal faecal specimens were collected in sterile containers and stored at -80 °C, and microbial DNA was extracted from the faecal samples using a Soil DNA Kit (M5635-02, Omega Bio-Tek, Norcross, GA, USA). The extraction was performed with a 96-well nucleic acid extraction instrument (NanoMagBio S-96, Thermo Fisher, USA). All procedures were carried out strictly following the instructions provided with the kit. The concentration of DNA was measured using a NanoDrop NC2000 ultraviolet spectrophotometer (Thermo Fisher, USA), and the quality of the DNA was assessed via 1% agarose gel electrophoresis. The DNA concentration was adjusted, the working solution was stored at 4 °C, and the storage solution was stored at -20 °C.

### Polymerase chain reaction (PCR) amplification

The 16 S rRNA gene variable regions were amplified using a PCR thermal cycler (Applied Biosystems 2720, USA). PCR was conducted according to the instructions provided in the Q5@ High-Fidelity DNA Polymerase Kit (New England Biolabs, Beverly, MA, USA). The sequences of primers used were as follows: ACTCCTACGGGAGGCAGCA (Forward) and GGACTACHVGGGTWTCTAAT (Reverse), which was synthesized by Personal Biotechnology Co., Ltd., Shanghai, China. The amplified products were purified using gel recovery, and the target bands were excised for further purification. The purified samples were quantified using the Quant-iT PicoGreen dsDNA Assay Kit (P7589, Invitrogen, Carlsbad, CA, USA).

### Library construction and sequencing

DNA fragments with overhanging ends were repaired, and a single nucleotide ‘A’ was introduced at the 3’ end of the repaired DNA fragments. Tagged adapters were incubated with the DNA fragments by ligase to facilitate their interaction. After purification and enrichment of DNA fragments, the libraries were quantified using the Quant-iT PicoGreen dsDNA Assay Kit (P7589, Invitrogen, Carlsbad, CA, USA). All libraries were normalized to 10 nM, mixed in equal volumes, and gradually diluted to a concentration of 4–5 pM before being subjected to high-throughput sequencing.

### The profiles of amino acids and acylcarnitine in neonates

Within a week after birth, heel blood samples were collected from the neonates, dripped onto custom filter paper (Fenghua Bioengineering Co., Ltd., Guangzhou, China), and air-dried to obtain blood spots, which were stored in a refrigerator at 4–8 °C to control humidity. Using an automatic puncher (DBS 220, Fenghua Bioengineering Co., Ltd. Guangzhou, China), 3.2 mm diameter dried blood spots were obtained. The levels of amino acids and acylcarnitines in the blood spots were subsequently determined using the NeoBase™ Non-derivatized MSMS Kit (Perkin-Elmer, Waltham, MA, USA). Quantitative analysis of the analytes was conducted in a tandem mass spectrometry analysis system (Waters-ACQUITY TQD, USA), and the results were determined via comparison with known concentrations of stable isotope internal standards.

### 16 S rRNA gene sequencing data analysis method

The DADA2 method in QIIME2 (version 2019.4) was used for primer trimming, quality filtering, denoising, merging, and chimeric removal to obtain a denoised amplicon sequence variant (ASV). Alpha diversity analysis was performed using QIIME2 (version 2019.4), R language (version 3.2.0), and the ggplot2 package, with the Kruskal‒Wallis test used to discern differences in indices between the hyperbilirubinemia neonates and control individuals. Beta diversity analysis was conducted using R language, the vegan package, and the Bray‒Curtis statistical algorithm for nonmetric multidimensional scaling analysis (NMDS). Analysis of similarities (Anosim) was used to assess whether the inter-group differences were significantly greater than intra-group differences. Linear discriminant analysis effect size (LEfSe) was conducted using the Python LEfSe package, R language, ggtree package, and other analytical tools. The level of significance of differences between LEfSe analysis groups was assessed by using the Wilcoxon test. The phylogenetic tree was analysed by using the R language, ggtree package, and phyloseq package. In addition, the level of significance of the differences was measured by employing the Wilcoxon test. Random forest analysis was performed by using QIIME2. The principal coordinate analysis (PCoA) based on KEGG pathway information was performed by using the R language, vegan package, and ape. Permanova was used to assess the statistical significance of dissimilarities in PCoA based on KEGG pathway analysis between two groups. The association network was constructed by applying the sparse correlations for compositional data (SparCC) method.

### Statistical analysis

The statistical analysis was performed using SPSS 26.0 software. Descriptive statistics, including means and standard deviations, were used to present quantitative data for both groups. For normally distributed data, the t test or t’ test was applied, while non-normally distributed data were analysed using the Mann‒Whitney U test. Chi-square analysis was used for categorical data. Spearman’s rank-sum correlation coefficient was used to test the associations between intestinal microbiota and blood indices. A *p value* < 0.05 was considered to indicate statistical significance.

## Results

### Taxonomic composition at the phylum and genus levels

**Fig. 1:**
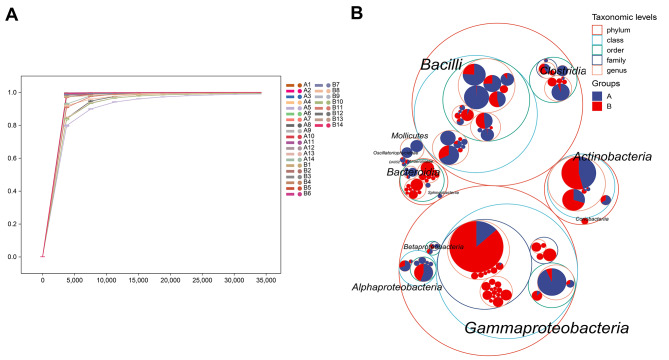
General description of the sequencing procedure. **(A)** The rarefaction curve based on Goods_coverage of Alpha diversity analysis. **(B)** Taxonomic tree of the two groups. The largest circles represent the phylum level, while the inner circles represent class, order, family, and genus. Group A: the hyperbilirubinemia group; Group B: the control group

**Fig. 2:**
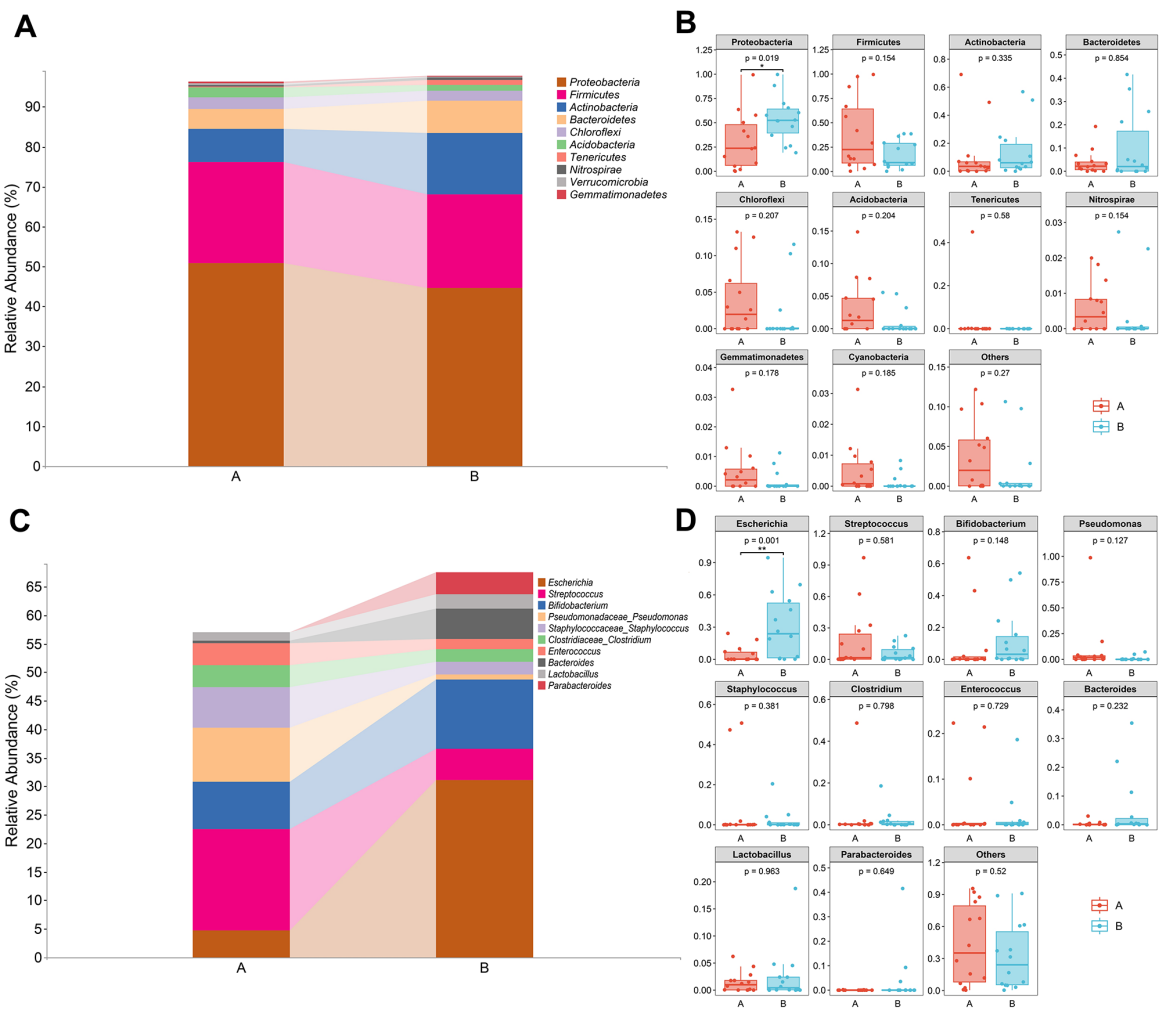
Taxonomic comparison of relative abundance at the phylum and genus levels. **(A)** Bar chart representing the data at the phylum level. **(B)** Differences in relative abundance at the phylum level between the two groups. **(C)** Bar chart representing the data at the genus level. **(D)** Differences in relative abundance at the genus level between the two groups. Group A: the hyperbilirubinemia group; Group B: the control group. * *p* < 0.05, ** *p* < 0.01

Twenty-eight samples were included in this sequencing. The rarefaction curve, which was generated based on the Goods_coverage of Alpha diversity analysis and species accumulation curve (Fig. 1A and S1), was flat, indicating that there was enough sequencing data to cover most bacteria and a sufficient number of sequences from the two groups. This allowed the requirements for further analysis to be met. Figure 1B shows a classification grade tree diagram depicting the whole classification of the two groups of samples. The histogram in Fig. 2A shows the top 10 phyla and their average proportions in the two groups. The two groups of intestinal microorganisms were roughly the same in terms of phylum and were composed mainly of *Proteobacteria*, *Firmicutes*, *Actinobacteria*, *Bacteroidetes*, and *Chloroflexi*. In addition, there were slight differences between the two groups regarding the proportion of bacteria. A statistical analysis, shown in Table 2, revealed that lower proportions of *Proteobacteria*, *Actinobacteria*, and *Bacteroidetes* were found in the hyperbilirubinemia group than in the control group, while *Firmicutes*, *Chloroflexi*, and *Acidobacteria* had greater proportions. The abundance of *Proteobacteria* was significantly lower in the hyperbilirubinemia group than in the control group (Fig. 2B). At the genus level, *Escherichia*, *Streptococcus*, and *Bifidobacterium* were the predominant taxa in the two groups (Fig. 2C). The results revealed that the proportion of *Escherichia* and *Bifidobacterium* was lower in the hyperbilirubinemia group than in the control group, while *Streptococcus*, *Pseudomona*s, and *Staphylococcus* demonstrated greater proportions (Table 3). The abundance of *Escherichia* was significantly lower in the hyperbilirubinemia group than in the control group (Fig. 2D). The phylogenetic tree mapping revealed the differences in the top 50 ASVs in the samples. Notably, the ASV_37570 sequence exhibited a significantly greater representation of *Escherichia* in the control group than in the hyperbilirubinemia group (Fig. S2).

**Table 2:** The average proportion of phylums

Phylum level	Groups (%)	
	Hyperbilirubinemia group	Control group
*Proteobacteria*	30.58	53.67
*Firmicutes*	38.45	17.20
*Actinobacteria*	11.36	14.02
*Bacteroidetes*	3.85	9.90
*Chloroflexi*	3.95	1.76
*Acidobacteria*	3.17	1.05
*Tenericutes*	3.24	0.01
*Nitrospirae*	0.59	0.38
*Gemmatimonadetes*	0.54	0.17
*Cyanobacteria*	0.51	0.12
Others	3.76	1.74

**Table 3:** The average proportion of genus level

Genus level	Groups (%)	
	Hyperbilirubinemia group	Control group
*Escherichia*	4.71	31.15
*Streptococcus*	17.77	5.47
*Bifidobacterium*	8.26	12.04
*Pseudomonas*	9.53	0.94
*Staphylococcus*	7.17	2.26
*Clostridium*	3.77	2.10
*Enterococcus*	3.91	1.88
*Bacteroides*	0.37	5.24
*Lactobacillus*	1.49	2.54
*Parabacteroides*	0.02	3.89
Others	42.98	32.46

### Results for alpha and beta diversity

**Fig. 3:**
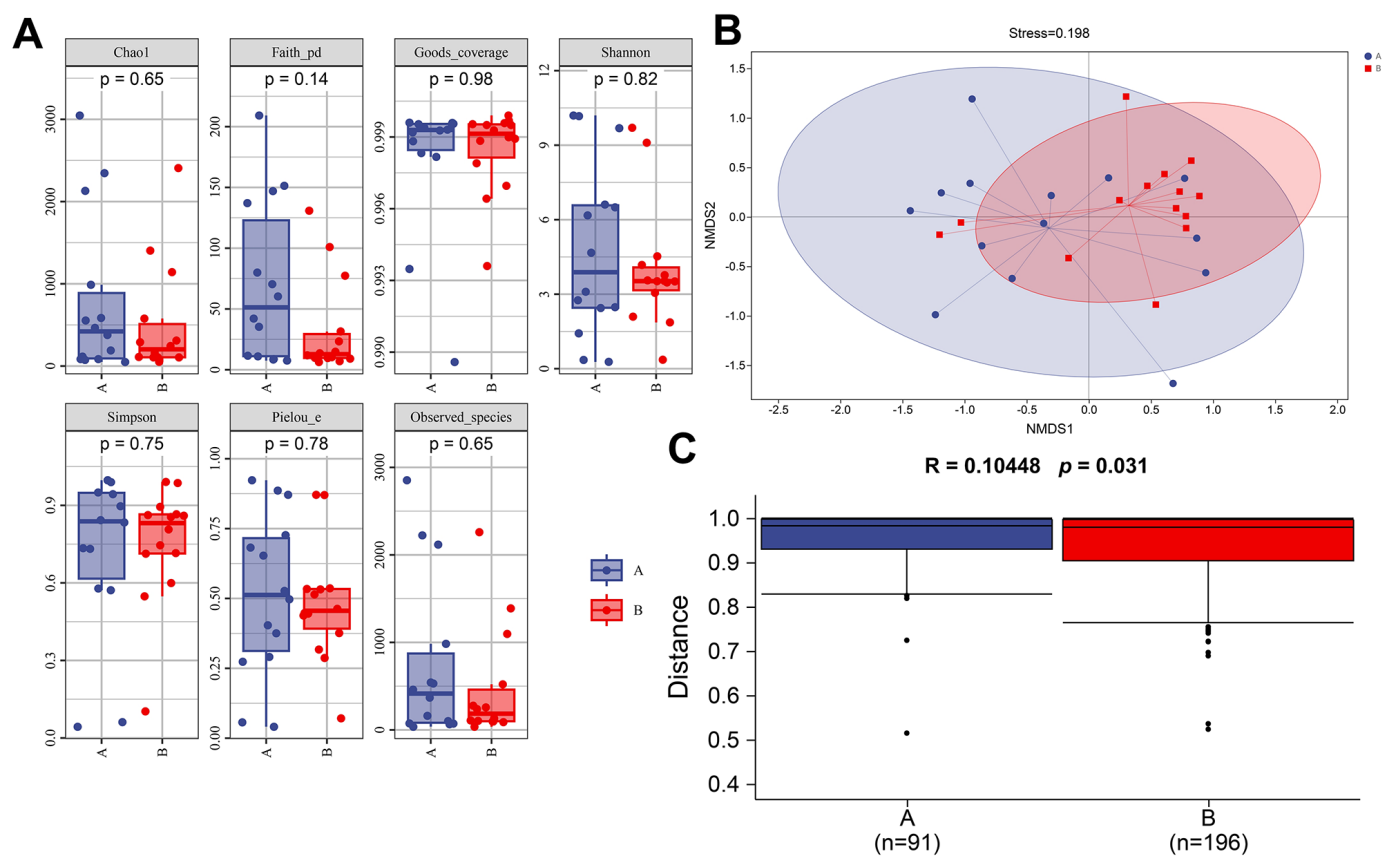
Alpha and Beta diversity between the two groups. **(A)** The Alpha diversity indices of the two groups. **(B)** Nonmetric multidimensional scaling analysis (NMDS) of the two groups. **(C)** Analysis of differences between the two groups using Anosim. Group A: the hyperbilirubinemia group; Group B: the control group

The two groups of Alpha diversity indices (Chao1, Faith_pd, Goods_coverage, Observed_species, Pielou_e, Shannon and Simpson) were slightly different, but the differences were not statistically significant (*p* > 0.05) (Fig. 3A). The Goods_coverage index observed for the two groups were the same, indicating that there was no significant difference in the proportion of bacteria that had not been detected in the samples. The Bray‒Curtis distance was calculated for NMDS. The calculated distance of each sample and the bacterial community structures were similar within the same group, while differences were observed between samples within different groups (Fig. 3B). The stress value calculated in the NMDS was 0.198, proving that the analysis results were reliable. We further used Anosim to test whether there were significant differences between the groups, and the R value was 0.10448, indicating that there was a greater difference between the groups than within the group (*p* = 0.031). This also indicated greater significant differences in the bacterial community structures between the two groups (Fig. 3C).

### Results of differential bacteria analysis

**Fig. 4:**
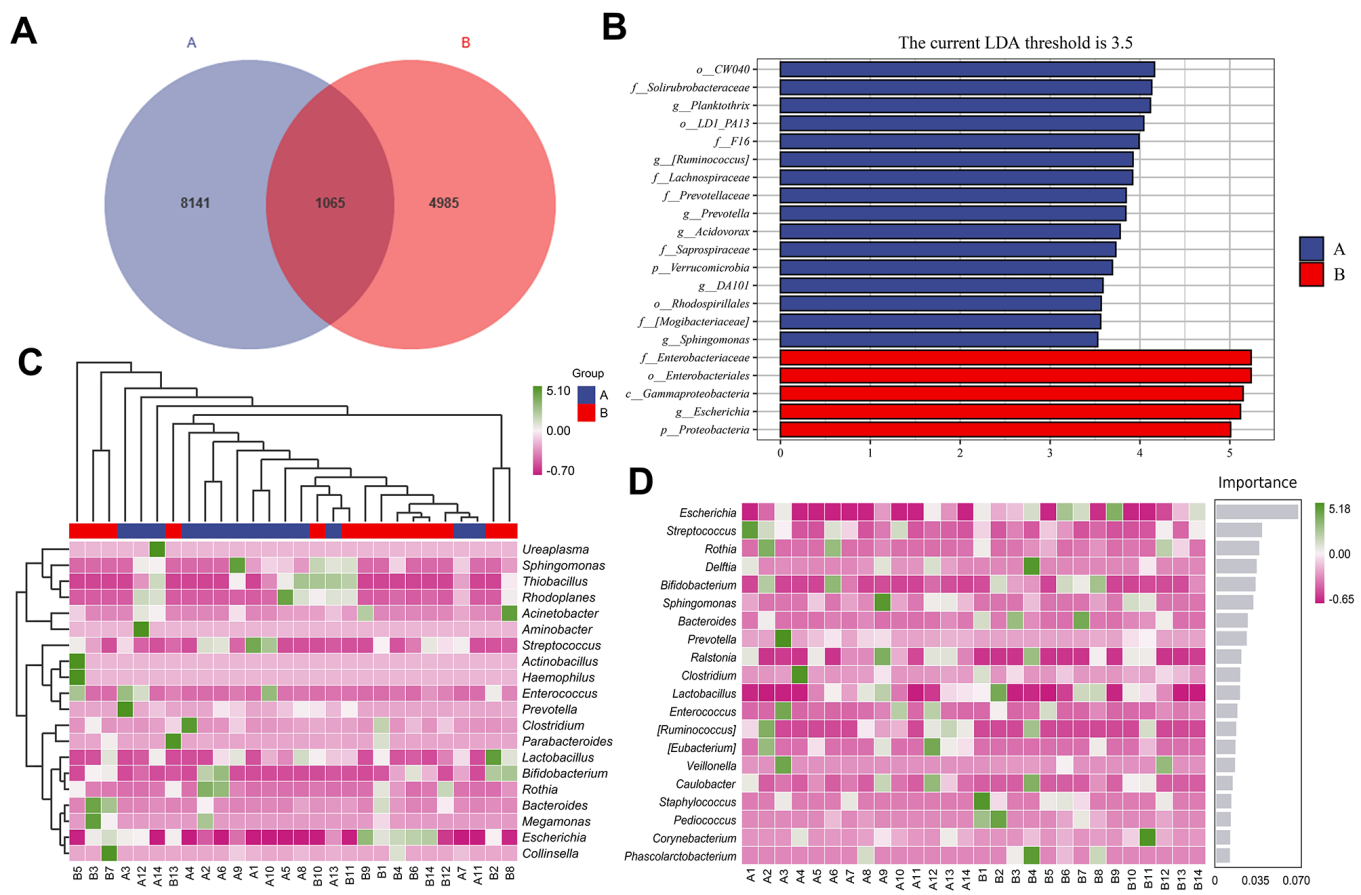
The results of bacterial difference analysis. **(A)** Venn diagram. **(B)** Histogram of LDA scores showing significant differences in microbe type and abundance between the two groups. The letters p, c, o, f, and g represent the taxonomic ranks at the phylum, class, order, family, and genus levels, respectively. **(C)** Heatmap of the bacterial composition at the genus level. **(D)** The analysis of random forests at the genus level. Group A: the hyperbilirubinemia group; Group B: the control group

**Fig. 5:**
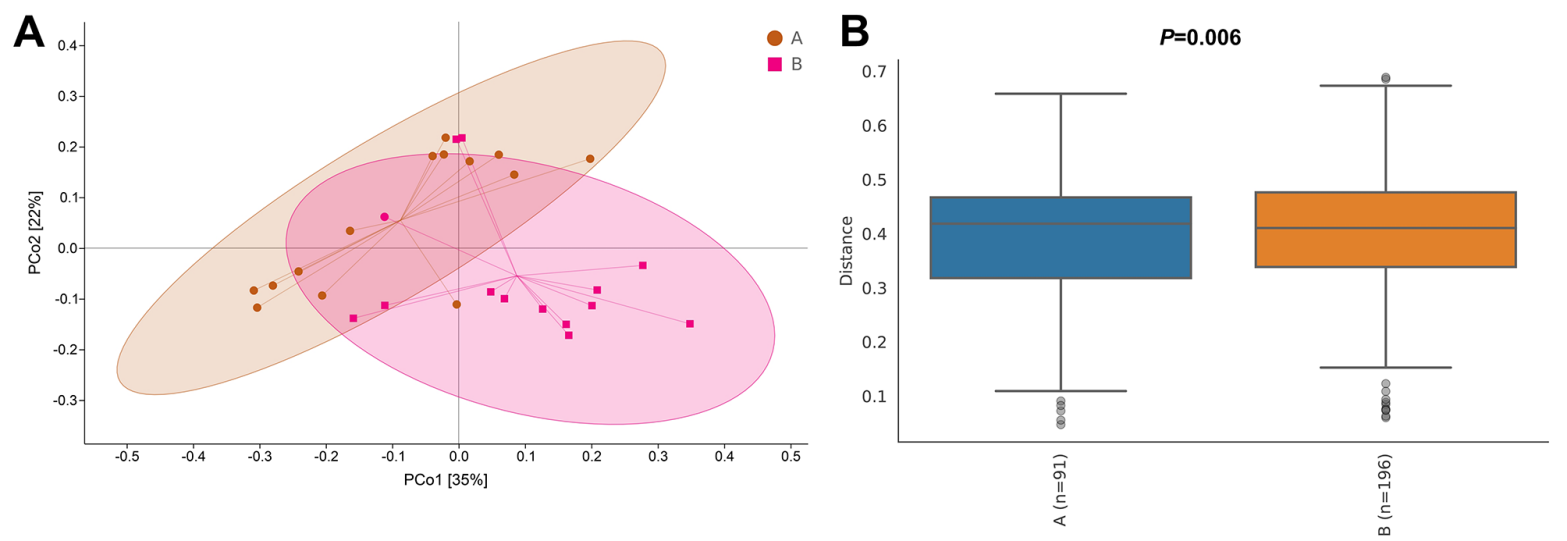
The differences between the two groups of metabolic pathway analysis. **(A)** Principal coordinate analysis (PCoA) based on the KEGG pathways of the two groups. **(B)** Analysis of differences between the two groups using Anosim. Group A: the hyperbilirubinemia group; Group B: the control group

**Fig. 6:**
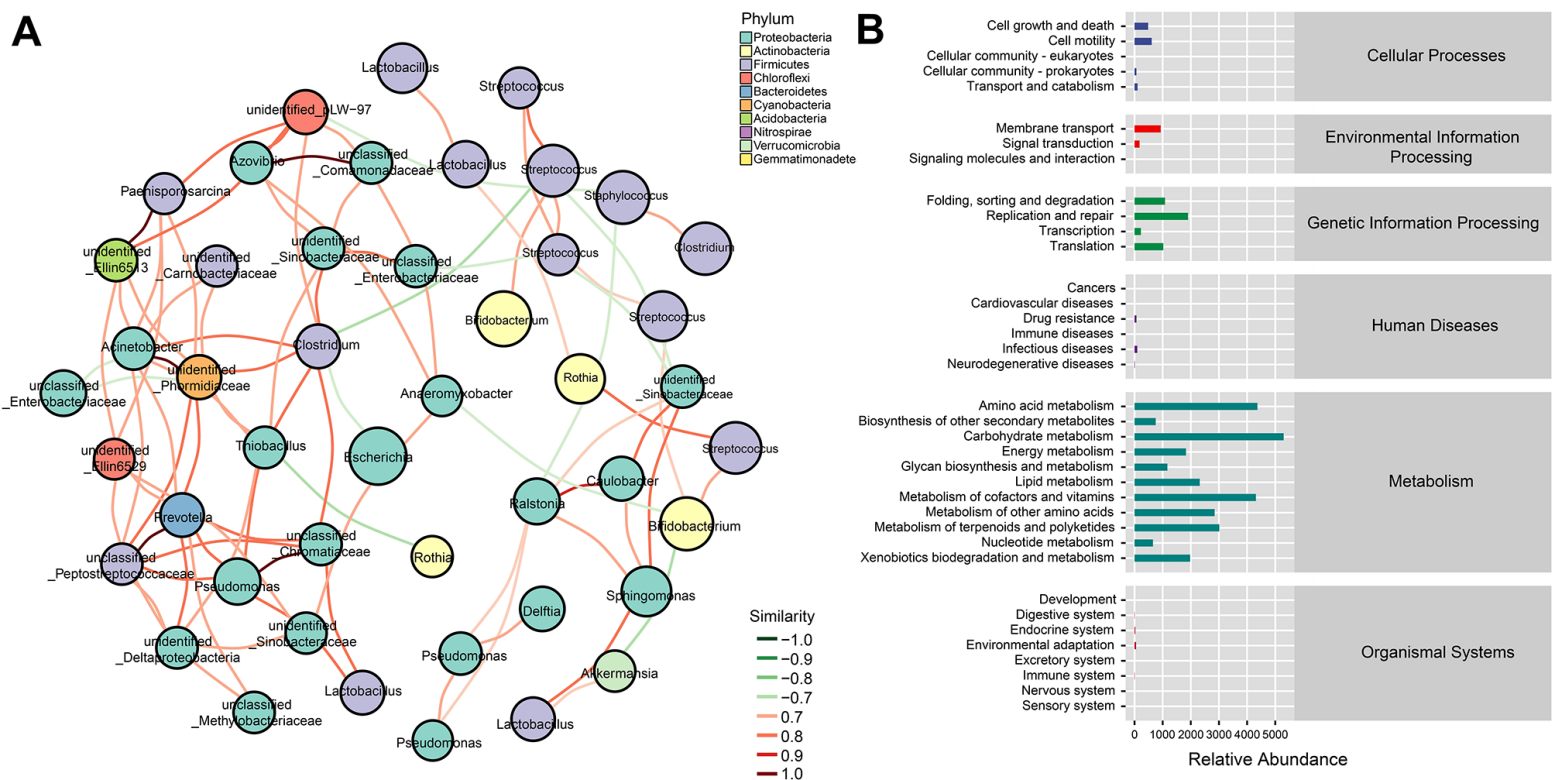
The results of metabolic pathway analysis. **(A)** The results of network analysis. **(B)** Metabolic pathway based on the KEGG pathway database

A Venn diagram was generated to visualize ASV abundance (Fig. 4A), and the results showed that 28 patients produced 14,191 ASVs, 8141 (accounting for 57.37%) of which were specific to the hyperbilirubinemia group, while 4985 (accounting for 35.13%) and 1065 cross-sequences were found in the control group and two groups, respectively (accounting for 7.5%). LDA effect value histograms and cladograms were generated after screening for bacterial groups with significant differences between groups using LEfSe (Fig. 4B and S3). The results indicated that the abundances of *Gammaproteobacteria* and *Enterobacteriaceae* were significantly lower in the hyperbilirubinemia group than in the control group, while the abundances of *Ruminococcus* and *Sphingomonas* were greater than those in the control group. A heatmap was generated to compare the bacterial composition among the samples at the genus level, and the results showed the distribution of the top 20 genera between the two groups in the various samples and the trend in the abundance distribution of the species in each sample (Fig. 4C). The diagram of the random forest plot illustrates the distribution of genus abundance, specifically highlighting the top 20 distinct genera in each sample. In descending order of importance, the genera within the sample exhibited a decreasing trend, *Escherichia*, *Streptococcus*, and *Rothia* were the top three genera (Fig. 4D). The gene families (KEGG homologous genes) of the two groups of bacteria were analysed by employing PICRUSt2. After obtaining the abundance data of the metabolic pathways, the functional disparities, based on the KEGG signaling pathways, were evaluated by using the Bray‒Curtis distance algorithm. The PCoA revealed statistically significant differences between these two groups (Fig. 5A-B). The network diagram revealed enrichment of bacteria belonging to the phylum *Proteobacteria*, indicating strong interconnections with other bacteria (Fig. 6A). Based on the KEGG database annotation, an abundance diagram depicting functional channels was constructed, and the abundance of genes associated with metabolic functions was relatively high (Fig. 6B).

### Comparison of amino acid and acylcarnitine contents in the peripheral blood of neonates

Studies show that the increased levels of blood amino acids and acylcarnitine can be observed in hyperbilirubinemia neonates [13]. Therefore, we further examine whether the changes of intestinal microbiota of hyperbilirubinemia neonates are associated with the levels of blood amino acids and acylcarnitine. Tandem mass spectrometry (TMS) was used to measure the levels of amino acids and acylcarnitines in the peripheral blood of neonates in the two groups to determine whether there were differences between the two groups. Among the 11 detected amino acids, citrulline (Cit), arginine (Arg), ornithine (Orn), and valine (Val) had significantly greater levels in the hyperbilirubinemia group than in the control group (*p* < 0.05). No statistically significant difference was found between the two groups regarding the levels of other types of amino acids (*p* > 0.05). In addition, among the 20 types of acylcarnitine, the levels of free carnitine (C0), hexanoylcarnitine (C6), octanoylcarnitine (C8), and stearoylcarnitine (C18) in the hyperbilirubinemia group were significantly greater than those in the control group (*p* < 0.05). There were no statistically significant differences in the levels of the other types of acylcarnitines between the two groups (Table 4).

**Table 4:** Differences in amino acid and acylcarnitine profiles between the two groups

Analytes (umol/L)	Hyperbilirubinemia group		Control group		Statistic value	*p* Value
	Mean	SD	Mean	SD		
**Amino acids**						
Citrulline (Cit)	37.41	26.23	23.11	32.28	Z = -2.205	0.027
Phenylalanine (Phe)	52.50	25.05	48.00	20.93	t = 0.516	0.611
Methionine (Met)	55.34	38.89	34.95	27.30	Z = -1.562	0.118
Tyrosine (Tyr)	154.25	84.22	113.47	46.42	Z = -1.241	0.215
Alanine (Ala)	232.34	70.15	215.95	70.17	t = 0.618	0.542
Leucine (Leu)	113.80	108.67	120.80	70.35	Z = -0.138	0.890
Arginine (Arg)	59.64	62.09	20.63	23.77	Z = -2.022	0.043
Ornithine (Orn)	244.51	158.55	132.33	95.21	Z = -2.068	0.039
Glycine (Gly)	311.39	169.84	323.17	141.15	t = -0.200	0.843
Valine (Val)	163.66	46.87	118.91	21.11	Z = -2.803	0.005
Proline (Pro)	125.77	102.65	136.30	71.90	Z = -0.368	0.713
**Acylcarnitine**						
Free carnitine (C0)	30.15	26.26	17.95	5.48	Z = -2.481	0.013
Acetyl-l-carnitine (C2)	14.42	12.02	14.23	7.70	Z = -0.414	0.679
Propionyl carnitine (C3)	1.45	1.58	1.26	0.73	Z = -0.207	0.836
Butylcarnitine (C4)	0.27	0.19	0.19	0.05	Z = -0.943	0.345
Isovalerylcarnitine (C5)	0.14	0.23	0.07	0.04	Z = -0.515	0.606
Tiglylcarnitine (C5:1)	0.05	0.04	0.03	0.03	Z = -1.129	0.259
Hexanoylcarnitine (C6)	0.08	0.03	0.05	0.02	t = 3.042	0.005
Methylglutarylcarnitine (C6DC)	0.07	0.03	0.06	0.04	t = 0.351	0.729
Octanoylcarnitine (C8)	0.10	0.06	0.06	0.02	Z = -2.431	0.015
Decenoylcarnitine (C10:1)	0.06	0.06	0.05	0.02	Z = -0.093	0.926
Lauroylcarnitine (C12)	0.06	0.02	0.05	0.03	t = 0.916	0.368
Dodecenoylcarnitine (C12:1)	0.10	0.05	0.07	0.04	Z = -1.607	0.108
Myristoylcarnitine (C14)	0.12	0.07	0.10	0.06	t = 1.041	0.307
Myristoleylcarnitine (C14:1)	0.07	0.05	0.08	0.05	Z = -0.323	0.746
Palmitoylcarnitine (C16)	1.10	1.36	1.25	1.25	Z = -0.046	0.963
Decanoylcarnitine (C10)	0.30	0.48	0.46	0.75	Z = -0.531	0.596
Palmitoleylcarnitine (C16:1)	0.09	0.07	0.10	0.06	Z = -0.256	0.798
Stearoylcarnitine (C18)	0.88	0.25	0.67	0.14	Z = -2.277	0.023
Oleylcarnitine (C18:1)	0.89	0.72	0.95	0.48	Z = -0.023	0.982
Linoleylcarnitine (C18:2)	0.19	0.27	0.17	0.10	Z = -0.467	0.640

### Correlations between the intestinal microbiota and blood indicators

**Fig. 7:**
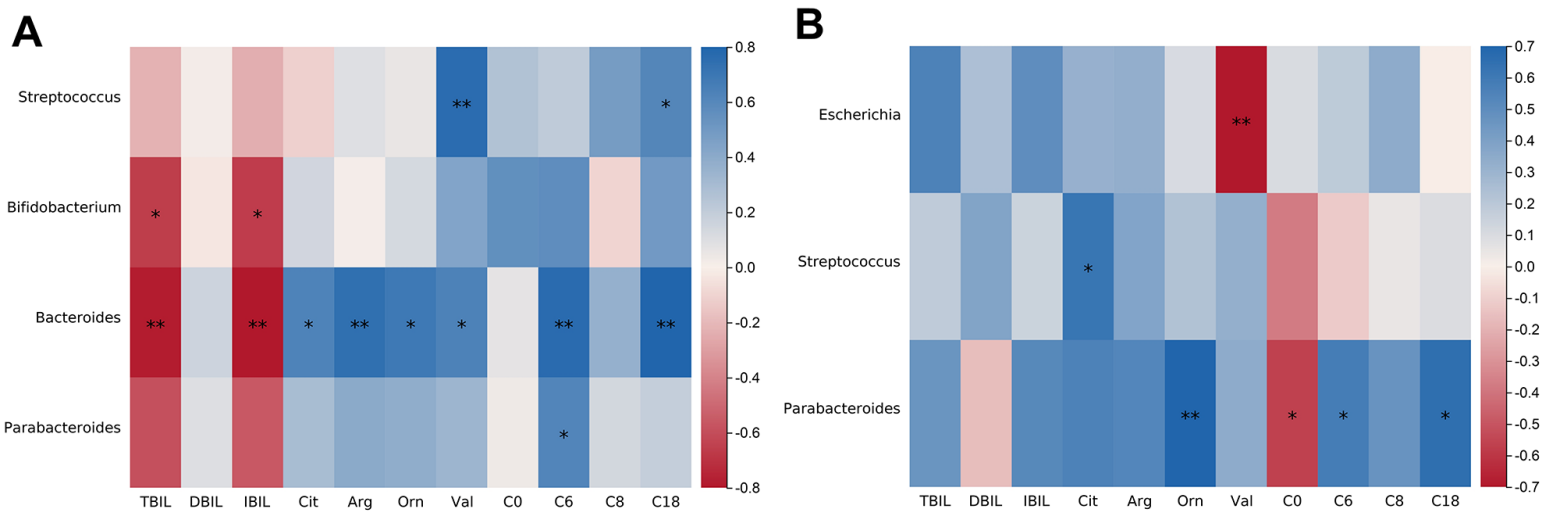
Correlations between the intestinal microbiota at the genus level and blood indices **(A)** The hyperbilirubinemia group. **(B)** The control group. A blue colour on the heatmap indicates a positive correlation, while a red colour indicates a negative correlation. The darker the colour is, the greater the correlation coefficient. * *p <* 0.05, ** p < 0.01

In the hyperbilirubinemia group, the levels of TBIL and IBIL exhibited significant inverse correlations with *Bacteroides* and *Bifidobacterium*, while the levels of Cit, Arg, Orn, Val, C6 and C18 exhibited significant positive correlations with *Bacteroides* (Fig. 7A). In the control group, the levels of Orn, C6, and C18 exhibited a significant positive correlation with *Parabacteroides *abundance, the level of Cit exhibited a significant positive correlation with *Streptococcus*, and the level of Val exhibited a significant inverse correlation with *Escherichia* (Fig. 7B).

## Discussion

This study has showed that the structures of the neonatal intestinal microbiota did significantly differ between the hyperbilirubinemia group and the nonhyperbilirubinemia group. *Gammaproteobacteria* and *Enterobacteriaceae* exhibited decreasing abundances, while *Ruminococcus* and *Sphingomonas* exhibited increasing abundances in hyperbilirubinernia group. The control group exhibited a greater percentage of *Escherichia* and *Bifidobacterium*, whereas the hyperbilirubinemia group exhibited a greater percentage of *Enterococcus* and *Streptococcus*. Profiles of amino acid and acylcarnitines in peripheral blood indicated that the levels of Cit, Arg, Orn, C6, C8, C18 and C0 in hyperbilirubinemia group were greater than those in the control group. In the hyperbilirubinemia group, blood bilirubin levels were negatively correlated with *Bacteroides* and *Bifidobacterium* abundances.

The intestinal microbiota is regarded as another ‘organ’ in the human body that plays a crucial role in various aspects of life beginning in the early stage of life, including nutrition, metabolism, and immune maturation [14]. In the hyperbilirubinemia group, the proportions of *Proteobacteria, Actinobacteria* and *Bacteroidetes* were lower than those in the control group, while the proportions of *Firmicutes, Chloroflexi* and *Acidobacteria* were greater. Imbalances in the intestinal microbial community, such as in *Firmicutes* and *Actinobacteria*, are considered contributory factors to jaundice [1]. Alpha diversity analysis in our study indicated that there were no significant differences in the overall richness or diversity of the intestinal microbiota between the two groups. These findings are somewhat consistent with most of the currently published data [1], suggesting that the diversity of the intestinal microbiota is not directly correlated with the degree of elevated bilirubin. Beta diversity analysis revealed significant differences in the intestinal microbiota structure between the hyperbilirubinemia group and the control group, indicating a certain degree of dysbiosis in the intestinal microbiota of the hyperbilirubinemia group. Comprehensive analysis of the results of our study revealed no significant differences in general information, such as sex, age, gestational age, weight, or Apgar score, between the hyperbilirubinemia group and the control group, which effectively excluded relevant factors that could interfere with the results of the intestinal microbiota. In the present study, the abundances of *Escherichia* and *Bifidobacterium* were lower in the hyperbilirubinemia group than in the control group, while the abundances of *Enterococcus* and *Streptococcus* were greater, suggesting that a discernible imbalance is potentially associated with elevated bilirubin levels. Based on the microbial member relationships, network analysis revealed that *Proteobacteria* was the most abundant and closely associated with other bacteria. We postulate that *Proteobacteria* may exert a pivotal influence on the composition of the intestinal microbiota; however, further investigations are imperative to substantiate this conjecture.

Neonates with hyperbilirubinemia are prone to liver dysfunction during disease, which can cause disturbances in amino acid metabolism. The early detection of amino acid levels is highly clinically important. Previous studies have indicated that elevated levels of Cit, Arg, Orn, isoleucine and proline reflect abnormal amino acid metabolism in neonates with jaundice [15]. In this study, the blood levels of Cit, Arg, Orn, and Val in neonates in the hyperbilirubinemia group were greater than those in the control group. Orn and Cit are two important metabolic products of Arg in the liver that are involved in the urea cycle [16]. We assume that hepatic injuries in hyperbilirubinemia neonates cause the increased level of amino acids. Val, one of the seven nonessential amino acids, is a branched-chain amino acid associated with liver repair. In this study, neonates with hyperbilirubinemia exhibited higher levels of Val compared to the control group, which is consistent with the findings of previous literature [17]. We hypothesize that the body initiates a self-repair mechanism. Acylcarnitine is an important product of organic acid and fatty acid metabolism. The results of this study indicate that the hyperbilirubinemia group primarily exhibited elevated levels of medium-chain fatty acids (MCFAs, including C6 and C8), long-chain fatty acids (LCFAs, including C18), and free carnitine (C0). The primary function of free carnitine is to facilitate the breakdown of fatty acids for energy within cells [18]. It is speculated that the liver cells of neonates with hyperbilirubinemia stimulate the body’s self-protective mechanism to increase the energy supply during the process of breaking down, binding, and transporting bilirubin. Various studies have demonstrated that MCFAs exert a significant impact on the *Firmicutes*-to-*Bacteroidetes* ratio in the murine gut, leading to downregulation of the relative abundance of *Proteobacteria* [19]. The elevated levels of C6 and C8 in the hyperbilirubinemia group may have contributed to the decreased relative abundance of *Proteobacteria*. It has been reported in the literature that the ability of the neonate liver to excrete bilirubin is insufficient, leading to temporary intrahepatic bile stasis [2]. Therefore, we speculate that the elevation of LCFAs in the hyperbilirubinemia group is associated with bile stasis.

The present study also revealed a significant negative correlation between the levels of TBIL and IBIL in neonates and the abundance of *Bacteroides* and *Bifidobacterium*, which is consistent with contemporary research findings [1, 20]. *Bacteroides* play an important role in maintaining the structure and function of the intestinal mucosal barrier [21]. *Bifidobacterium*, as a normal part of the human intestinal microbiota, have immunomodulatory, antitumour and anti-inflammatory effects and are closely related to the production of DBIL [20]. Therefore, we postulate that the elevated bilirubin levels observed in neonates with hyperbilirubinemia may impact the colonization of *Bacteroides* and Bifidobacterium within the intestinal tract, leading to structural and functional impairments of the intestinal mucosal barrier. In addition, this could affect the production of DBIL and subsequent disruption of enterohepatic circulation, ultimately contributing to impaired bilirubin metabolism and its accumulation in the bloodstream. This intricate process underscores potential interactions between these factors. In addition, the levels of Cit, Arg, Orn, Val, C6 and C18 in the hyperbilirubinemia group were significantly positively correlated with *Bacteroides*. We speculate that the metabolism of these amino acids and acylcarnitine was closely regulated by *Bacteroides*. However, due to the limitations of 16 S rRNA gene sequencing technology, it was not possible to identify which bacterial species played a role.

In recent years, great progress has been made in research on the intestinal microbiota among neonates with hyperbilirubinemia, immune diseases, and biliary atresia. This progress provides a substantial collection of fundamental theoretical bases for treatment with probiotics. Proper treatment could be provided based on the indicators gained by detecting intestinal microbiota, amino acids, and acylcarnitine, thereby reducing the mortality rate among neonates and allowing neonates to progress well soon. There are several limitations in 16 S rRNA gene sequencing techniques, as they tend to be performed on the basis of the diversity of the bacterial community, which prevents identification of the types of bacteria that play roles in the metabolism of bilirubin. We will further analyse the specific mechanism underlying the interaction between intestinal microecology and serum bilirubin levels by employing metagenomics sequencing and metabolomics.

## Conclusions

In conclusion, the findings of the present study indicated that associations could be observed among the levels of intestinal microbiota, blood amino acids, and blood acylcarnitines in neonates with hyperbilirubinemia. This could provide a certain theoretical basis for clinical diagnosis and treatment. The mechanism of the specific types of bacteria involved in the regulation of amino acid and acylcarnitine metabolism will be further analysed.

### Electronic supplementary material

Below is the link to the electronic supplementary material.

Supplementary Material 1

Supplementary Material 2

Supplementary Material 3

Supplementary Material 4

## Data Availability

Raw sequence data are available in the write full meaning of SRA database under accession number PRJNA1069669 (https://www.ncbi.nlm.nih.gov/sra/PRJNA1069669).

## References

[1] You JJ, Qiu J, Li GN, Peng XM, Ma Y, Zhou CC. The relationship between gut microbiota and neonatal pathologic jaundice: a pilot case-control study. Front Microbiol. 2023;;14:1122172. . 10.3389/fmicb.2023.1122172 PMC10060978 37007464

[2] Par EJ, Hughes CA, DeRico P. Neonatal hyperbilirubinemia: evaluation and treatment. Am Fam Physician. 2023;;107(5):525–34. . 37192079

[3] Najati N, Gharebaghi MM, Mortazavi F. Underlying etiologies of prolonged icterus in neonates. Pak J Biol Sci. 2010;;13(14):711–4. . 10.3923/pjbs.2010.711.714 21848064

[4] Henny-Harry C, Trotman H. Epidemiology of neonatal jaundice at the University Hospital of the West Indies. West Indian Med J. 2012;;61(1):37–42. . 22808564

[5] Qin J, Li R, Raes J, Arumugam M, Burgdorf KS, Manichanh C. A human gut microbial gene catalogue established by metagenomic sequencing. Nature. 2010;;464(7285):59–65. . 10.1038/nature08821 PMC3779803 20203603

[6] Colella M, Charitos IA, Ballini A, Cafiero C, Topi S, Palmirotta R. Microbiota revolution: how gut microbes regulate our lives. World J Gastroenterol. 2023;;29(28):4368–83. . 10.3748/wjg.v29.i28.4368 PMC10415973 37576701

[7] Felix KM, Tahsin S, Wu HJ. Host-microbiota interplay in mediating immune disorders. Ann N Y Acad Sci. 2018;;1417(1):57–70. . 10.1111/nyas.13508 PMC5889363 28984367

[8] Vítek L, Zelenka J, Zadinová M, Malina J. The impact of intestinal microflora on serum bilirubin levels. J Hepatol. 2005;;42(2):238–43. . 10.1016/j.jhep.2004.10.012 15664250

[9] Koníčková R, Jirásková A, Zelenka J, Lešetický L, Štícha M, Vítek L. Reduction of bilirubin ditaurate by the intestinal bacterium Clostridium perfringens. Acta Biochim Pol. 2012;;59(2):289–92. . 22540115

[10] Tabibian JH, O’Hara SP, Trussoni CE, Tietz PS, Splinter PL, Mounajjed T. Absence of the intestinal microbiota exacerbates hepatobiliary disease in a murine model of primary sclerosing cholangitis. Hepatology. 2016;;63(1):185–96. . 10.1002/hep.27927 PMC4670294 26044703

[11] Ding J, Ma X, Han L, Zhao X, Li A, Xin Q. Gut microbial alterations in neonatal jaundice pre- and post-treatment. Biosci Rep. 2021;;41(4):BSR20210362. . 10.1042/BSR20210362 PMC8150162 33860293

[12] Maisels MJ, Bhutani VK, Bogen D, Newman TB, Stark AR, Watchko JF. Hyperbilirubinemia in the newborn infant > or = 35 weeks’ gestation: an update with clarifications. Pediatrics. 2009;;124(4):1193–8. . 10.1542/peds.2009-0329 19786452

[13] McCarthy ME, Oltman SP, Baer RJ, Ryckman KK, Rogers EE, Steurer-Muller MA. Newborn metabolic Profile Associated with Hyperbilirubinemia with and without Kernicterus. Clin Transl Sci. 2019;;12(1):28–38. . 10.1111/cts.12590 PMC6342241 30369069

[14] Chen K, Yuan T. The role of microbiota in neonatal hyperbilirubinemia. Am J Transl Res. 2020;;12(11):7459–74. . PMC7724329 33312382

[15] Schmid-Rüter E, Feist D (1976). Hypermethioninemia in the differential diagnosis of infantile obstructive jaundice (author’s transl). Monatsschr Kinderheilkd (1902).

[16] Zhang W, Zheng J, Zhang J, Li N, Yang X, Fang ZZ. Associations of serum amino acids related to urea cycle with risk of chronic kidney disease in Chinese with type 2 diabetes. Front Endocrinol (Lausanne). 2023;;14:1117308. . 10.3389/fendo.2023.1117308 PMC10018121 36936143

[17] Zeng S, Wang Z, Zhang P, Yin Z, Huang X, Tang X. Machine learning approach identifies meconium metabolites as potential biomarkers of neonatal hyperbilirubinemia. Comput Struct Biotechnol J. 2022;;20:1778–84. . 10.1016/j.csbj.2022.03.039 PMC9027383 35495115

[18] Sasenick J, Miller M, Rastogi D, Morrissey M, Rastogi S. Carnitine supplementation increases serum concentrations of free carnitine and total acylcarnitine in preterm neonates: a retrospective cohort study. JPEN J Parenter Enter Nutr. 2023;;47(6):746–53. . 10.1002/jpen.2535 37345267

[19] Zhou S, Wang Y, Jacoby JJ, Jiang Y, Zhang Y, Yu LL. Effects of medium- and long-chain triacylglycerols on lipid metabolism and gut microbiota composition in C57BL/6J mice. J Agric Food Chem. 2017;;65(31):6599–607. . 10.1021/acs.jafc.7b01803 28704610

[20] Akagawa S, Akagawa Y, Yamanouchi S, Teramoto Y, Yasuda M, Fujishiro S. Association of Neonatal Jaundice with gut dysbiosis characterized by decreased Bifidobacteriales. Metabolites. 2021;;11(12):887. . 10.3390/metabo11120887 PMC8705620 34940645

[21] Martin-Gallausiaux C, Marinelli L, Blottière HM, Larraufie P, Lapaque N. SCFA: mechanisms and functional importance in the gut. Proc Nutr Soc. 2021;;80(1):37–49. . 10.1017/S0029665120006916 32238208

