# Supplementary figures and images for: Analysis of the intestinal microbiota and profiles of blood amino acids and acylcarnitines in neonates with hyperbilirubinemia
Source: BMC Microbiol. 2024 May 18;24:171. doi: 10.1186/s12866-024-03328-y (PMC11102171; doi:10.1186/s12866-024-03328-y)

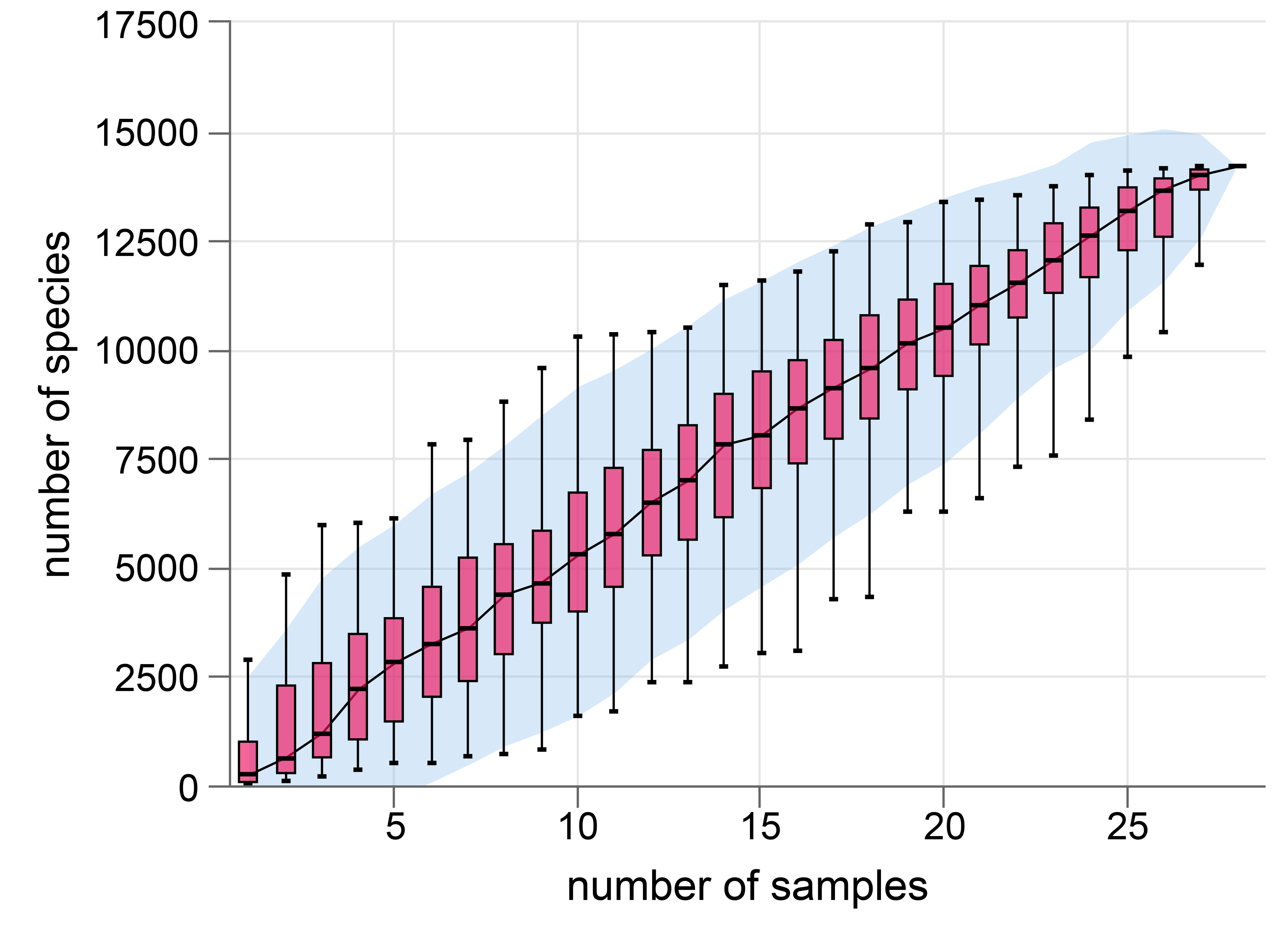

Supplement: Supplementary file 1 — Supplementary Material 1 [file 12866_2024_3328_MOESM1_ESM.jpg]

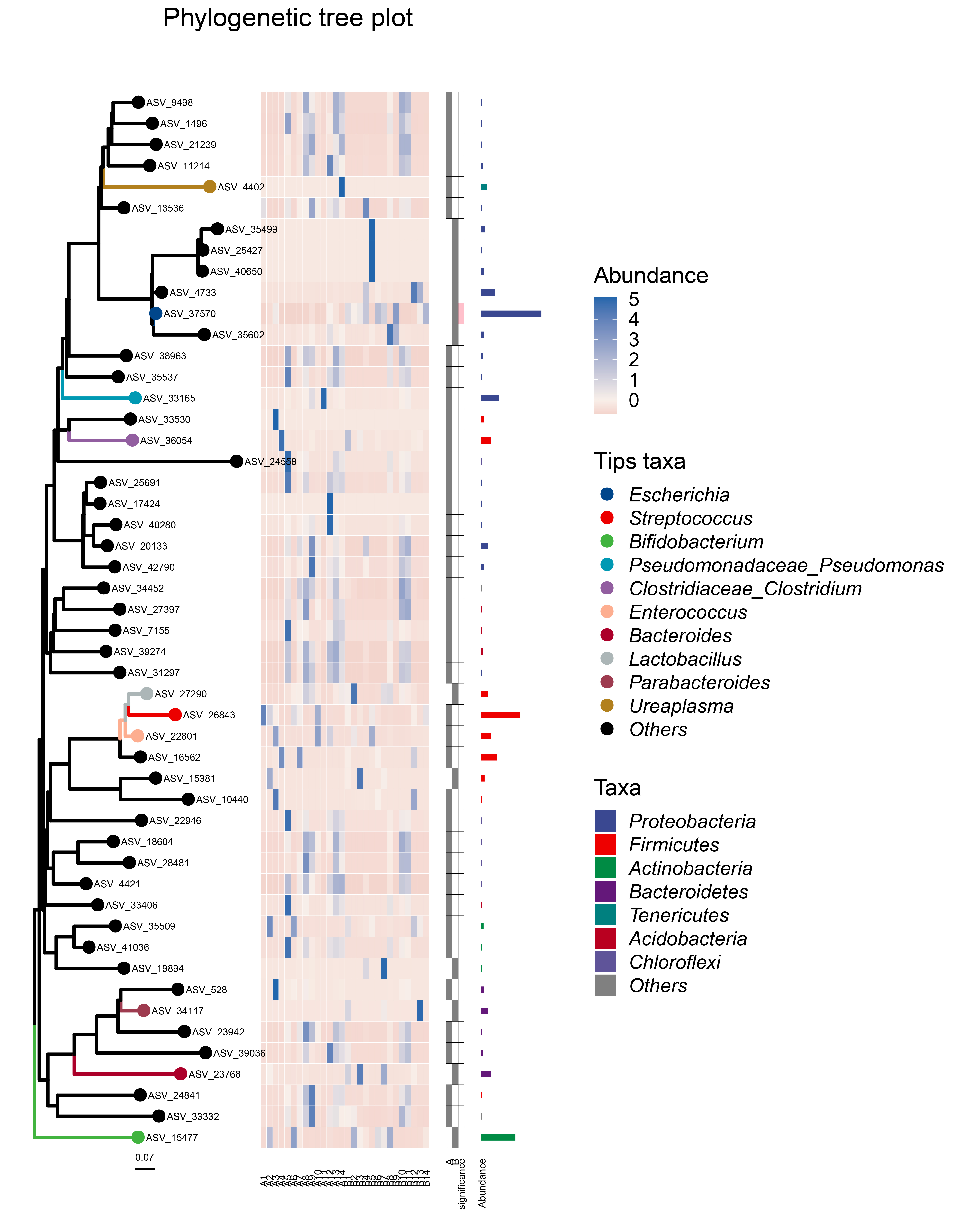

Supplement: Supplementary file 2 — Supplementary Material 2 [file 12866_2024_3328_MOESM2_ESM.jpg]

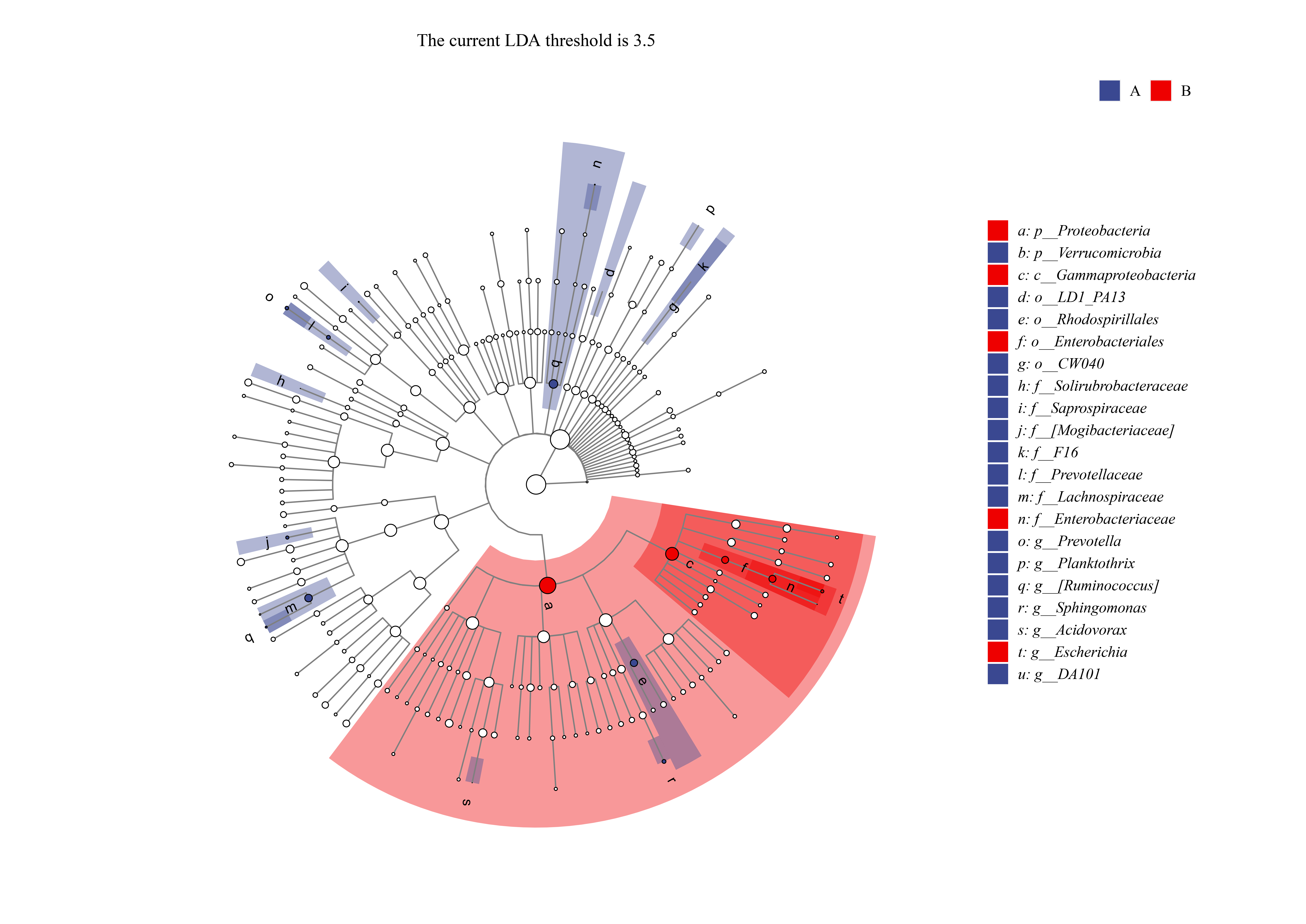

Supplement: Supplementary file 3 — Supplementary Material 3 [file 12866_2024_3328_MOESM3_ESM.jpg]
