# Supplementary material for: Analysis of the intestinal microbiota and profiles of blood amino acids and acylcarnitines in neonates with hyperbilirubinemia
Source: BMC Microbiol. 2024 May 18;24:171. doi: 10.1186/s12866-024-03328-y (PMC11102171; doi:10.1186/s12866-024-03328-y)
Supplement: Supplementary file 4 — Supplementary Material 4 [file 12866_2024_3328_MOESM4_ESM.doc]

**Fig S1** Species accumulation curve.

**Fig S2** The phylogenetic tree map. The figure illustrates the relative abundance of bacteria represented by different ASV sequences in each sample. Notably, *Escherichia* corresponding to the ASV_37570 sequence exhibits a significantly higher abundance in control group compared to the hyperbilirubinemia groups. Group A: the hyperbilirubinemia group, Group B: the control group.

**Fig S3** The cladogram generated by LEfSe.

The taxonomic branching diagram illustrates the hierarchical organization of major taxa within a sample community, ranging from phylum to genus (from the inner circle to the outer circle).
